# Supplementary material for: Are carbon emissions trading and green financial instruments synergistic? -Comprehensive quantitative research based on content analysis
Source: PLoS One. 2024 Mar 7;19(3):e0298601. doi: 10.1371/journal.pone.0298601 (PMC10919601; doi:10.1371/journal.pone.0298601)
Supplement: S1 Data — (ZIP) [file pone.0298601.s001.zip › Data/地级市Z_F工作报告.zip!/╔┬╬≈/░▓┐╡╩╨/└┤╫╘╣1⁄2╓┌║┼í╛┬φ┐╦╩2╛▌═°í┐_2016.docx]

各位代表：

  现在，我代表市人民政府向大会作工作报告，请予审议，并请市政协委员和列席人员提出意见。

  一、2015年和“十二五”工作回顾

  2015年，在市委的坚强领导和市人大、市政协的监督支持下，全市上下共同努力，圆满完成了全年目标任务。实现生产总值772.46亿元，增长12.3%。生产总值、第二产业增加值、全社会固定资产投资、公路客货运周转量增速和非公经济增加值占GDP比重五项经济指标列全省第一。财政一般公共预算收入30.8亿元，同口径增长13.2%；城镇居民人均可支配收入27191元，农村居民人均纯收入8196元，分别增长8.7%和9.8%；单位生产总值能耗下降4%。

  一是投资保持较快增长。全社会固定资产投资完成758.2亿元，增长25.2％。谋划多年的安康火电厂、旬阳、白河水电站和烟厂技改项目开工建设，28个产业项目建成投产。安平高速建成通车，安康机场、西成客专、阳安铁路复线、引汉济渭、安康中心港、洞河水库建设稳步推进，石紫岚、平利至镇坪、安康至白河、宁陕至两河二级路等12条489公里道路升等改造加快实施。

  二是循环产业提质增效。安康高新区升级为国家高新技术产业开发区，同时被认定为全国新型工业化富硒食品产业示范基地。全市规模以上工业增加值增长17.6%，增速全省第二。培育市级以上现代农业园区180个、农业产业化龙头企业124户，新型职业农民培育工作得到中省肯定；生猪、茶叶、魔芋等特色产业加快发展。旅游综合收入增长20.8%。新增限额以上商业企业136家，培育电商企业177户，社会消费品零售总额增长13.5%。

  三是新型城镇化建设加快。中心城市创建为国家卫生城市，市政建设完成投资17亿元。江滩公园二期、500个停车位、14个公交站、4座人行天桥建成，城东大桥、金州路综合改造、静宁市场、南正街和城区背街小巷改造顺利推进，东西二坝、西南、黄沟、长春片区棚户区改造启动实施。月河川道城镇带、石紫岚沿江生态经济带及县城、重点镇和新型社区建设加快。实施棚户区改造2.07万户，保障性住房开工2.79万套，农村居民进城入镇8.3万人。

  四是改革开放迈出坚实步伐。政府机构改革、镇村综合改革完成，事业单位分类改革、国有企业监管体制改革、公车改革有序推进。市县区政务服务中心实现全覆盖。文化、教育、医疗卫生改革和“百千万”人才工程深入推进。高新区科技创业孵化中心被确定为国家科技企业孵化器，岚皋、紫阳创建为省级电子商务示范县。财政金融改革创新成效明显，申报国家政策性金融扶贫试验示范区进展顺利，民生金融创新在全省推广，全市金融机构各项存款、贷款余额分别增长16.8%和15.4%。对外开放不断扩大，招商引资实际到位资金406亿元。

  五是社会事业加快发展。完成民生支出186.2亿元，增长12.3%。“十件实事”取得实效。新增城镇就业1.96万人，农村劳动力转移就业63.7万人。学前教育和高中教育普及率达95.95%和96.28%，贫困学生资助和营养餐实现全覆盖，中心城区新增学位1.2万个。实施县镇村卫生服务设施改扩建项目976个，市儿童医院建成。中心城区累计新增公交车160辆、出租车130辆，创造条件开通中心城区到恒口、瀛湖、关庙公交线路。环境保护力度加大，四项主要污染物削减任务完成，绿化造林66万亩，中心城区天然气入户率70%。科技创新能力增强，技术合同成交总额3200万元。培育规上文化企业45家，文化产业增加值增长11%。完成体育健身工程418个，市体育中心综合训练馆主体建成。专项扶贫投入29.36亿元，实现12.3万贫困人口脱贫。建成15个社区日间照料中心和100个农村幸福院。

  六是行政效能稳步提升。坚持把建设人民满意政府作为努力方向，推进简政放权，转变政府职能，公布市级部门权力清单和责任清单，对506项重点任务和民生实事向群众公开承诺。坚持向市人大及其常委会报告工作，向市政协通报情况，自觉主动接受监督；办理人大代表建议议案167件、政协委员提案246件。扎实推进政府系统党风廉政建设，查处群众身边的不正之风和腐败问题600件，党政纪处分751人。深入开展“三严三实”专题教育，严格执行改进作风规定，市级“三公”经费支出下降23%，审计全覆盖工作受到国家审计署肯定。其他各项工作也都取得了新的成绩。

  各位代表， 2015年各项任务的顺利完成，标志着“十二五”规划圆满收官。过去的五年，是我市经济发展提速增效、生态优势加快转化、社会事业蓬勃发展、群众生活显著改善的五年。五年来，我们紧紧围绕市三次党代会和市三届人大一次会议确定的目标任务，坚持走民生为本的循环发展之路，取得了一批填空白、蓄势能、补短板的重要成果，实现了经济、社会、文化和生态文明建设的全面进步。

  经济快速发展、综合实力显著增强。“十二五”规划提出的“四个翻番”和“六个跃升”目标基本实现。全市生产总值年均增长13.6％，2015年经济总量是2010年的2.4倍；规模工业增加值年均增长24.6%，县域工业集中区标准化厂房面积超过百万平方米；现代农业园区、特色产业发展、农业经营主体培育取得重大突破。财政一般公共预算收入是“十一五”的3.06倍，年均增长18.5%，高于全省6.2个百分点，支持发展和改善民生的能力显著增强。

  基础设施极大改善、瓶颈制约有效缓解。全社会固定资产投资累计完成2531亿元，年均增长25.7%。在交通、水利、电力、环保、电信、市政等领域建成了一批重大项目。汉江综合整治、东坝防洪工程等一批重大基础项目加快实施，防灾减灾能力增强。旬阳330千伏和6个110千伏输变电工程建成，湖南至酒泉800千伏特高压输电工程安康段启动建设，农村电网改造实现全覆盖。建成十天、安平高速和西康铁路复线，全市高速公路达577公里，公路通车总里程2.3万公里，居全省第二，长期制约发展的交通短板正在成为竞争新优势。

  经济结构不断优化、特色产业快速发展。三次产业结构调整到12.4:55.3:32.3。产业集约化程度提高，“三区两园”和“飞地经济”园区成为重要增长极。科技支撑能力增强，获中省科学技术奖37项，申请专利1560件，技术合同成交额1.2亿元。工业化进程加快，完成工业投资570亿元，净增规模工业企业190户，产值过亿元企业达292户。新型材料、装备制造、富硒食品总产值分别从73.3亿元、4.4亿元、24.5亿元跃升到354亿元、85亿元、234亿元。服务业增加值年均增长10.4％。农业基础地位更加稳固，粮食产量保持在80万吨以上，富硒茶园扩大至70万亩，生猪规模养殖量全省第一，魔芋产量占全国七分之一。建成8个4A级景区，旅游接待人数、总收入分别是“十一五”的4.4倍和5.2倍。

  城乡面貌深刻变化、新型城镇化加速推进。全市城镇化率44.3％，五年提高近10个百分点。城市基础设施建设投入153亿元，是“十一五”的2.6倍。中心城市建成区面积39.5平方公里，城市人均道路面积16平方米、绿地12.6平方米，污水处理率86%、生活垃圾无害化处理率99%。房地产投资212亿元，建设保障性安居工程16.5万套，实施棚户区改造6.8万户。创建国家卫生县城5个，实现省级卫生县城、园林县城全覆盖。建设县乡示范路872公里、通村示范路8923公里、连户路8911公里、农村客运站点626个，城乡面貌发生了深刻变化。

  生态优势加快转化、可持续发展能力不断增强。坚持“护水、增绿、治污、移民、兴业、富民”同步推进。治理水土流失4500平方公里，绿化造林326.4万亩，森林覆盖率提高到65％，居全省第一。建成21个县城污水和垃圾处理场，实施汉江沿线18个重点镇污水、垃圾处理项目，汉江水质持续稳定达标。关停“两高”企业300余家，淘汰落后产能92万吨标准煤，万元GDP能耗由0.7911吨标准煤下降到0.7595吨标准煤。生态立市、循环发展形成共识，循环产业成为经济增长的重要支撑，为可持续发展奠定了坚实基础。

  重点改革纵深推进、开放型经济稳步发展。国家主体功能区建设试点示范深入推进。国有企业资产管理得到加强。教育改革和资源整合全面推进。医药卫生体制改革取得重大进展，探索形成的多个医改模式引起良好反响。食品药品监管体制改革顺利推进，实现市县镇村监管网络全覆盖。经营性文化事业单位改制取得实效。引进民营资本发展体育产业。农村土地经营权、集体林权制度改革、殡葬改革等进展顺利。财政金融改革稳步推进，政府统一协调管理的“大金融”格局基本形成。对外开放层次、领域和质量不断提高，招商引资取得显著成效，累计签订招商引资合同项目1070个，实际到位资金1462亿元。

  社会事业全面发展、人民群众获得感不断提升。完成民生支出698亿元，是“十一五”的3.46倍。城乡居民收入年均增长13.2%和15.6%。整合294亿元资金投入扶贫，减少贫困人口44.28万人，避灾扶贫搬迁50.16万人，安全饮水工程惠及172万人。城镇累计新增就业8.5万人次。社会保障基本实现全覆盖。教育事业全面发展，创建国家义务教育均衡发展县5个、省级“双高双普”县7个、教育强县3个。完成医疗卫生项目投资13亿元，各类医疗卫生机构增加到3190家，新农合参合率99%以上。文化事业和文化产业加快发展，创建全国文化工作先进县2个、省级文化先进县5个，文化产业增加值实现近10倍增长。实施镇村（社区）体育健身工程1780个。人口计生、老龄、慈善、红十字、残疾人、妇女儿童、民族宗教、外事侨务等事业加快发展。社会治理不断强化，健全公共突发事件应急机制，安全生产形势稳定，“六五”普法深入推进，信访工作得到加强，9个县创建为全省平安建设先进县，全市社会治安满意率连续五年全省领先。各级政府积极推进廉政和作风建设，工作效率和服务水平明显提高。

  五年的成绩来之不易，需要我们共同珍惜。五年的发展实践启示我们：向全面建成小康社会目标迈进，党的领导是根本保证，必须更加自觉地向中央基准看齐，坚决贯彻中、省和市委决策部署；加快发展是关键所在，必须坚持循环发展、追赶超越不动摇，持续保持全省领先的发展速度；生态环境是宝贵资源，必须坚持保护与开发并重，促进全民共享绿色福利；改革创新是持久动力，必须深化改革开放、实施创新驱动，推动发展迈向中高端水平；改善民生是根本目的，必须坚持以脱贫攻坚总揽全局，让人民生活更加美好；改进作风是必然要求，必须干字当头，勇尽责、敢担当，发扬愚公移山精神，夺取全面建成小康社会的新胜利。

  各位代表，过去五年所取得的成就，是省委、省政府和市委坚强领导，市人大、市政协鼎力支持的结果，凝聚着全市人民的智慧和汗水。这里，我谨代表市人民政府，向全市广大干部群众、各位代表、各位委员和社会各界人士，向离退休老干部、中省驻安单位、驻安解放军、武警和消防官兵、政法干警，向所有关心支持安康发展的各界朋友，致以崇高敬意和衷心感谢!

  回顾过去五年的工作，我们也清醒认识到存在的困难和问题。经济总量小，产业结构层次不高，资源环境约束趋紧，市场主体创新能力不强，实现新旧增长动力转换、推动经济转型升级还需付出艰辛努力；贫困人口比重大，城乡居民收入低，区域发展不平衡，生态建设和环境保护责任重大，实现脱贫攻坚和全面小康任务艰巨；转变政府职能，改进干部作风，建设人民满意政府还需持续用力。对此，我们一定直面问题不回避，勇于担当不退缩，切实有效加以解决。

  二、“十三五”发展的总体要求和目标任务

  “十三五”是全面建成小康社会的决胜期，也是建设美丽富裕新安康的关键期。全市改革发展的总体要求是：深入贯彻党的十八大、十八届三中、四中、五中全会和习近平总书记系列重要讲话精神，按照“四个全面”战略布局，落实“五大发展理念”，坚定不移走民生为本的循环发展之路，以追赶超越、富民兴安为核心，以发展升级、绿色崛起为主线，突出精准脱贫攻坚、循环产业发展、基础设施建设、民生保障改善和生态环境保护，协同推进新型工业化、新型城镇化、农业现代化、信息化和绿色化进程，开创美丽富裕新安康建设的新局面，实现全面建成小康社会奋斗目标。

  “十三五”发展的预期目标是：年均经济增速高于全省平均水平2个百分点，到2020年全市生产总值突破1450亿元，全社会固定资产投资年均增长15%以上，财政一般公共预算收入年均增长10%以上，城乡居民收入年均分别增长9%和11%以上，达到或超过全省平均水平，与全国全省同步进入全面小康社会。

  （一）优化经济社会发展布局，培育区域经济新的增长极。以国家主体功能区建设试点示范为带动，加快重点区域率先突破，把安康国家高新区建成现代化城市新区、高新技术产业聚集区和循环经济发展示范区，把恒口示范区建成全省镇级小城市综合改革试验区和城乡一体化发展示范区，增强县域工业集中区、现代农业园区和“飞地经济”园区承载能力。协同推进月河川道城镇与产业经济带、石紫岚沿江生态经济带建设。支持县区发挥特色优势，争先进位创一流。加强和深化与沿江区域合作，推动汉江生态经济带进入国家战略层面。

  （二）构建绿色低碳循环产业体系，推动产业向中高端迈进。突出新型工业化发展，推动形成两个产值过千亿元、三个产值过百亿元的产业集群。加快转变农业发展方式，做优做强特色高效富硒产业，建成全国富硒食品产业基地，打造“中国硒谷”地域品牌。发挥旅游资源优势，打造全国知名的全域旅游发展示范区。加速升级现代服务业，建设全国有影响的区域物流中心和健康养生基地。实施“互联网+”行动计划，推动产业组织和商业模式创新，加快三次产业融合发展，构建绿色低碳循环产业体系。

  （三）强力推进精准扶贫，坚决打赢脱贫攻坚战。以脱贫攻坚统揽全局，强化政府责任,引领市场、社会协同发力,构建大扶贫工作格局。拓宽脱贫攻坚投入渠道，坚持财政扶贫、金融扶贫、社会扶贫相结合，采取异地搬迁、发展产业、劳务创业、生态建设、发展教育、健康扶贫、社保兜底“七个一批”脱贫举措，确保2020年全市现行标准下的贫困人口全部脱贫、贫困县全部摘帽。围绕农业发展、农民增收、农村美丽，加大基础设施投入，构建现代农业发展体系，建设美丽乡村、文明家园。

  （四）建成区域交通枢纽，全面提升基础保障能力。围绕建设区域综合交通枢纽，加快高铁、公路、机场、航运等交通建设，构建内外畅达、互联互通的现代交通运输体系。建成全省一级物流节点，建设区域物流中心。推进重大水利工程、“五小水利”工程、防洪减灾工程和节水型社会建设。加强智能电网建设，推进农网升级。围绕打造智慧安康，构建宽带信息基础网络、物联网络和智慧城市信息化公共服务平台，提升绿色循环发展的基础保障能力。

  （五）加快推进新型城镇化，打造“秦巴明珠”。统筹城镇空间、规模、产业三大结构，规划、建设、管理三大环节，改革、科技、文化三大动力，生产、生活、生态三大布局，政府、社会、市民三大主体，构建“一心一带、七城多点”的新型城镇化格局。强化中心城市辐射带动作用，建设区域中心城市和国家级绿色生态新城，打造“秦巴明珠”。推进月河川道城镇带及县城、重点镇、新型社区和美丽乡村协调发展。加快城乡公共服务均等化，有序推进农业转移人口市民化，2020年全市城镇化率达到55%。提升城市综合管理水平，建成省级文明城市、环保模范城市和国家森林城市、园林城市。

  （六）厚植生态优势，建设美丽安康。坚持生态、环保、节能优先，加快生态优势向发展优势转化，建成国家生态文明综合改革试验区。加强生态环境综合治理和国土空间造林绿化，“十三五”末森林覆盖率达到68%。完善汉江水质保护长效机制，建成沿江沿河城镇、社区垃圾和污水处理设施，确保汉江水质稳定达标。加强重点污染物防治、农村面源污染治理和地质灾害区治理，建设生态安全体系。坚持铁腕治污防霾，持续改善大气质量。全面推行循环低碳发展模式，大力发展节能环保产业。集约节约利用国土资源，严守耕地保护红线。大力弘扬生态文化，推动生产生活方式绿色化，保护好天蓝、地绿、水净的美丽家园。

  （七）实施创新驱动战略，全面深化改革开放。强化全面创新，突出科技创新，推动管理创新，注重协同创新，形成大众创业、万众创新的浓厚氛围，推动新技术、新产业、新业态蓬勃发展。深化经济体制及财税、投融资体制等改革创新，推进资源配置市场化。加快农村改革、扶贫开发体制机制创新以及社会治理、社会事业改革。提升对外开放与经济协作水平，积极融入丝绸之路经济带和长江经济带，发展更高层次的开放型经济。实施人才强市战略，创新人才培养、引进、激励机制，引进培养一批科技创新领军人才和创新团队。

  （八）保障和改善民生，提升群众幸福指数。持续加大民生投入。始终把就业摆在优先位置，促进城乡居民稳定增收。扩大优质教育资源覆盖面，推行13年免费教育。实施“健康安康”工程，深化医药卫生体制改革，完善食品药品安全治理体系，加快发展体育事业，构建覆盖全生命周期的“大健康”服务体系。完善社会保障体系，健全社会救助体系。加强城镇棚户区和农村危房改造，改善城乡居民住房。加快文化事业和文化产业发展，健全现代公共文化服务体系。积极应对人口老龄化，推动养老服务多元发展。加强和创新社会治理，全面建设法治安康、平安安康，让全市人民生活更加幸福。

  根据中、省和市委决策部署，市政府在深入调研论证、广泛征求意见的基础上，制定了《安康市国民经济和社会发展第十三个五年规划纲要（草案）》，提请本次大会审议。我们将逐年分解实施并报告执行情况。

  三、2016年任务和重点

  2016年是实施“十三五”规划的第一年。主要预期目标是：生产总值增长10.5%，财政总收入和一般公共预算收入分别增长8%和12%，城乡居民收入分别增长9%和11%，当年脱贫12.2万人、减少贫困村200个，实现“十三五”良好开局。

  （一）坚持稳中求进，确保经济快速增长

  扩大有效投资。实施200个市级重点项目，全社会固定资产投资增长16%。重点加快机场、火电、水电、烟厂技改等重大项目建设，开工建设平镇高速，力争启动安岚高速。实施110个产业项目，建成钒氮合金二期、南水汽配、睿智建材、荣氏饮品、博灏环保、电池级硫酸锰等产业项目。加快西成客专、阳安铁路复线及石紫岚、旬小、安康至白河、平利至镇坪、宁陕至两河二级路、石泉至宁陕高速连接线及4座汉江大桥、安旬天然气输送工程等基础项目建设。大力推进汉江综合整治、洞河水库、月河补水等水利项目以及瀛湖治理、“两厂（场）”建设等生态项目。加大棚户区改造、教育卫生、文化设施、养老服务等民生项目建设力度。着力优化投资结构，增强社会投资活力，积极推进PPP模式，健全以产业引导基金等方式为主的财政支持体系。

  激活消费需求。多渠道增加城乡居民收入，增强居民消费能力。实施质量强市战略，支持企业适应高品质、个性化消费需求，积极开发适销对路的新产品，加快技术、业态创新，扩大有效供给。促进商贸物流业提质增效，加强城市商圈、各类专业市场建设，高标准实施城区金州路、兴安路改造提升。加快天贸物流城二期、江南物流园区、巨川富硒现代产业园、今东现代物流园、天一机械市场、江北一级货运站、秦巴明珠商贸城等项目建设。激发文化、教育、体育、信息、住房和绿色等领域消费需求，打造生态旅游、健康养老、家政服务等安康品牌。优化城乡市场环境，健全城乡市场流通体系，保持物价水平总体稳定。

  深化对外开放。坚定不移地实施开放战略，坚持“招大、引强、选优”，加大点对点招商、产业链招商、以商招商、驻地招商力度，引进更多核心竞争力强、带动力大的国内外知名企业。组织企业、产品“走出去”，推动地方特色产品更广泛、更深入地走向全国乃至世界。积极融入、主动接受关天、成渝、江汉经济圈辐射带动，务实推进与友好城市、高等院校、科研机构合作。千方百计扩大出口，加快发展外向型经济。进一步优化投资环境，加强社会诚信体系建设，狠抓招商项目落地，确保招商引资到位资金420亿元。

  （二）实施“四大工程”，推动产业转型升级

  实施园区提升工程。大力推进“三区两园”和“飞地经济”园区管理模式、投融资机制创新，加快完善基础设施和公共服务配套项目，推动建立创新创业支持平台。坚持集约节约发展，提高项目准入门槛，依据投资强度、税收、就业等实行差别化供地，加快产业、项目向园区集聚。安康高新区围绕高端化、配套化、集聚化，进一步强化配套、打造商圈、聚集人气，加快培育一批高新技术产业，规划建设一批高端商贸中心，支持银行金融机构和非银行金融机构向高新区转移；完善创业孵化园和“众创空间”服务功能，培育一批创新型小微企业；加快新型材料、现代物流、先进制造、富硒食品、健康养老产业发展，打造一批创新型高端产业集群。瀛湖旅游区围绕建设全省文化旅游产业基地，加快旅游综合服务区、金螺岛和翠屏山庄升级改造，促进旅游、文化、创意相融合，设施、服务、管理相匹配，发挥对安康全域旅游的带动作用。恒口示范区围绕加快市域副中心建设，开展好镇级小城市综合改革试验，加快恒力装备制造产业园、沐尚温泉旅游开发、古镇美食文化街区等建设。飞地经济园区加强基础建设和招商引资，在项目、政策和资金上更多倾斜支持，促进企业落地；相关县区要切实加强选商选资，集约节约利用“飞地”资源，更多引进高端项目。县域工业集中区重点培育优质企业，带动主导产业突破，培育汉滨五里、旬阳生态、汉阴月河、平利工业集中区4个产值过百亿园区。加快现代农业园区发展，注重创新发展模式、提升科技含量，注重与二、三产业融合发展，注重向质量效益型转变，带动农业增效和农民增收。

  实施工业技改升级工程。坚持引进应用先进技术与提升自主研发能力相结合，实施一批重大技改升级工程，支持企业应用新技术、新设备，打造智能工厂和数字化车间，积极创建智慧企业，淘汰落后产能，壮大新型工业实力。立足水资源优势，加快富硒水、纯净水、矿泉水产业发展。实施“互联网+”战略，大力培育新产业、新业态，促进移动互联网、大数据、云计算、物联网等信息技术与传统产业跨界融合，扶持电子商务与网络金融服务、3D打印、无人机应用等产业。扶持劳动密集型企业发展，让群众在家门口挣钱致富。加大企业帮扶力度，促进中小微企业和个体工商户转型升级，着力培育30户规上工业企业、5户产值过5亿元企业。

  实施农业富硒品牌带动工程。加强富硒产业组织化、标准化和市场化建设，加快部省市共建中国富硒产业研究院，完善富硒产品检测检验体系，建立富硒产品质量标准。实施“一县十镇百园”生态富硒农业示范工程，做强做优生猪、富硒茶、魔芋、核桃、富硒粮油等特色优势产业，整合富硒茶叶品牌，推动建立富硒魔芋国家标准。提升蚕桑、烟草、中药材、蔬菜等传统产业。重点支持规模经营主体和产业融合项目，发展壮大产业化龙头企业、专业合作社、专业大户等新型农业经营主体，培育新型职业农民1000人。加强农田水利等农业基础设施建设，实施测土配方施肥和农药化肥减量使用，强化农产品质量安全监管。严格落实耕地保护制度，稳定粮食生产。

  实施现代服务业提质增效工程。加快生态旅游业发展，推进全域旅游，启动南宫山5A景区创建，支持每个县区集中力量至少打造1个精品景区。大力发展乡村旅游，加快“十镇百村”美丽乡村建设；完善旅游要素和服务体系，整合资源打造全市综合旅游服务平台；加强旅游环境综合整治，倡导诚信服务、优质服务、文明服务；深化与周边城市合作，加强旅游营销宣传，提升影响力和知名度；借鉴临潼《长恨歌》的演艺机制和模式，推进旅游与文化融合发展，延伸产业链，提高附加值。突出金融服务、现代物流、电子商务、人力资源服务、信息技术、商贸会展等重点领域，大力培育生产性服务业。提速发展健康养老、文化创意、家政服务、体育健身等生活性服务业，积极创建全省生态健康养老示范市和全国医养结合实验区，加快培育一批知名电子商务龙头企业，促进现代服务业聚集区提质增效。

  （三）围绕生态宜居，加快新型城镇化进程

  加快完善城镇功能。做美中心城市，实施“一江两岸”整体改造建设，完善休闲旅游功能，提升文化品位；对江南城区实行最严格的开发管控措施，设定公用场地、公共设施、绿化空间等标准，达不到标准的项目一律不得审批；重点推进棚户区改造，加强公共设施提升，推进消防设施规划建设，加快社会服务、金融商贸、文化教育、医疗卫生和居住等功能北迁。加快月河川道城镇带建设，抓好县城、省级重点镇、县域副中心和20个标准化社区、旅游扶贫示范村建设，推动产城融合，壮大县域经济。坚持集约节约用地，拓宽城镇建设融资渠道，鼓励社会资本参与市政设施建设和经营。统筹城镇地上、地下设施建设，改造完善道路、管廊、线网等设施，推广“海绵城市”建设经验。大力发展公共交通，完善道路微循环系统。推动房地产转型发展，加快由单一地产向商住、旅游、养老等综合地产转变，促进刚性需求与改善性需求齐头并进。加强文化体育、医疗卫生、就业服务、社保经办和便民服务设施建设，增加城镇基本公共服务供给，让群众在家门口也能分享城镇化成果。在城镇建设中更加注重以自然为美，融入好山好水好风光；更加注重传承历史文脉、彰显地域特色；更加注重提高市民文明素养、打造城市精神，树立开放文明的良好形象。

  提升城镇管理水平。完善人口管理和服务体系，全面实施居住证制度，鼓励长期农民工举家进城落户。加快智慧城市建设，推进平安城市、智慧交通、城市光网建设，提升城镇管理数字化、精细化、规范化水平。强化城镇规划管理，创新规划理念和方法，增强规划的科学性和严肃性。理顺中心城市管理体制，坚持市区一体，合理界定市、区权责，夯实主体，分工协作，提高管理效能。开展“道路提升、环境整治、缓堵保畅”专项治理，坚持完善设施、教育引导与依法严惩相结合，加强交通行为、市容市貌规范管理，重点整治机动车、电动车等各类车辆非法营运、超载超速、乱停乱放、任意穿行、不守交规和流动商贩滥占公共场地摆摊设点、城区燃放烟花爆竹等行为。开展中心城区“三横两纵”道路精细化管理示范创建活动，实施城镇、社区居民文明提升行动计划，巩固“双创”成果，推进国家园林城市、健康城市和省级文明城市、环保模范城市创建，提升城镇文明度、宜居度、美誉度。坚持依法治市，深入推进城市管理和执法体制改革，加快培养一批懂城市、会管理的干部，完善城镇治理体系，提高综合治理能力。

  （四）推进改革创新，增强发展动力活力

  深化重点领域改革。积极推进供给侧结构性改革，提高供给体系质量和效益。开展降低企业成本行动，清理和规范涉企行政事业收费，推进流通体制、电价市场化等改革，逐步精简归并“五险一金”，创造良好环境，减轻企业负担，促进企业达产增效。实施高品质消费供给行动，提升工业产品质量，推动农产品绿色健康，发展精致生产，增加有效供给。加快政府职能转变，推行权力清单、责任清单管理模式，分类推进事业单位改革，提高政府行政效能。深化财税金融、投融资体制改革，积极推进“营改增”，创新金融产品和服务方式，推动直接融资发展，确保信贷增长18%以上。鼓励多种方式发展混合所有制经济，完善配套政策支持民营经济加快发展。完善经营性国有资产集中统一监管体系，优化国有资本布局和法人治理结构。积极推进价格机制改革，放开竞争性领域商品和服务价格。深化农村改革试点，着力在农村土地制度、集体产权制度、农业经营制度、农业支持保护制度、城乡发展一体化体制机制、农村社会治理制度改革、农村产权流转交易市场及服务平台建设等方面取得新进展。统筹推进文化、教育、卫生、生态文明建设、社会治理等各个领域改革，促进经济社会平稳健康持续发展。

  大力推进创新发展。把创新驱动作为加快发展的重大战略，实施“5+3”创新工程，培育全方位创新主体，着重在园区发展、制度创新、科技成果转化、激发大众创业和万众创新上取得新突破。加快推进科技创新，加大科技投入，实施重大科技专项，落实科技成果转化股权和分红激励政策，重点扶持10户科技创新示范企业、30户科技型“小巨人企业”、100户科技型小微企业。注重协同创新，促进技术、资本、信息、人才等创新要素有效整合与优化配置。创新财政产业资金投入方式，整合并统筹使用专项资金，设立产业发展基金、推行股权投资、实行以奖代补、财政贴息和政府购买服务，撬动更多社会资本投向产业。支持企业运用票据、债券等直接融资，积极培育企业上市。加强与中国农科院、药科大学、西安交大、陕西师大、西北农林科技大学、云南农大、安康学院等院校合作，建立机制灵活、互惠高效的产业技术创新战略联盟。组建安康创业风投，更大力度优化创新创业环境，推动大众创业、万众创新蓬勃发展。加大投资于人的力度，坚持培养、引进、激励并举，深入推进“百千万”人才工程，以更大的胸怀、更优惠的政策、更优越的条件，吸引更多的优秀人才和创新团队。

  （五）加强环境保护，推进生态文明建设

  完善汉江水质保护长效机制。坚持“源头严防、过程严管、后果严惩”，强化并完善“河长制”和网格化、流程化监管，发挥联席会议、目标考核和责任追究的制度作用，夯实汉江水质保护的主体责任。抓好汉江流域综合治理和水污染防治等项目实施，加强饮用水源地保护、地下水资源管理和重点江河断面水质监测。启动月河整治规划和丹治Ⅲ期工程，治理水土流失800平方公里。实施污水垃圾处理项目和工业点源治理项目，实现沿江重点镇(社区)污水垃圾处理设施全覆盖。发挥南水北调环境应急指挥处置中心作用，建成汉江干流6个水质自动监测站，提升环境应急处置能力。

  加大造林绿化和国土资源保护力度。实施新一轮“绿化安康”行动，大力推进全民植树、山地造林和城镇扩绿，突出江河两岸、城镇周边、公路沿线和重点景区，绿化造林50万亩，积极创建全国森林城市，实现省级生态县全覆盖。全面推进全省山林经济发展示范市建设。加强森林资源、野生动植物保护和自然保护区建设，加大秦巴山地乱采乱建治理力度，加快山地森林化步伐，维护生态安全和生物多样性。实行最严格的节约集约用地制度，强化土地综合利用，加大闲置土地处置力度，违反建设用地规定的一律依法收回，盘活存量土地，提高利用效率，确保稀缺资源优先用于重大基础设施、战略性新兴产业和民生项目。科学开发利用矿产资源，加强地质灾害防治，确保国土资源环境安全。

  强力推进治污防霾和节能减排。继续实施“治污降霾•保卫蓝天”行动计划，健全空气质量发布与预警机制。不断强化对燃煤锅炉、工地和道路扬尘、机动车尾气排放、秸秆焚烧、燃放烟花爆竹等主要污染源的监管和治理，有计划地淘汰粘土砖厂；实施企业环保设施运行在线监测，从严惩处违法违规行为，加快淘汰黄标车，坚决从源头上防治大气污染。加大“气化安康”、电能替代力度，加快气站、管网、充电桩等配套设施建设，提高清洁能源的覆盖面和普及率。开展企业节能低碳、绿色建筑和节能减排全民行动，加强工业点源和农业面源污染治理，严格控制对“两高”行业的信贷投放。实施城乡环境综合整治，抓好农村清洁能源应用。推行绿色、循环、低碳生产生活方式，促进生态环境共建共享。

  （六）办好民生实事，维护社会和谐稳定

  扎实推进精准扶贫。进一步加大投入，整合资源，调动力量，解决好扶持谁、谁来扶、怎么扶的问题，做到扶真贫、真扶贫、真脱贫，提高扶贫实效。深化“领导联村、部门包村、工作队驻村、第一书记入村、党员干部结对帮扶”工作机制，探索建立扶贫工作站，进一步摸清底数、找准问题、倒排时间，瞄准建档立卡贫困人口，坚持一村一策、一户一法，主攻579个重点贫困村整村脱贫帮扶工作。积极推进金融精准扶贫示范区建设，争取在全国率先使用扶贫再贷款。创新扶贫资源使用方式、开发模式和考评体系，完成财政专项扶贫投入20亿元。

  大力发展社会事业。坚持教育优先发展，全面实施学前教育三年行动计划、义务教育“全面改薄”和县城以上城区学校建设、普通高中改造等项目，扎实推进义务教育均衡发展“三大创建”，深入实施“三区一体化”、高效课堂、招生制度、教师轮岗交流等改革，加强青少年校外活动阵地建设。支持安康学院、安康职业技术学院转型发展。深入推进市县公立医院改革和县域医疗卫生综合改革试点，积极推广汉滨“茨沟模式”、宁陕一体化医疗服务、紫阳医院绩效分配制度改革等经验，加强执业医师和基层卫生计生人才队伍建设，提升公共卫生服务水平。实施机关事业单位养老保险制度改革，完善基本养老保险关系转移衔接政策，健全政府投入为主的医疗保险、大病救助、职工互助保险和社会保险相结合的医疗保险体系。加强城乡低保和农村五保工作。完善农村留守人员关爱政策，支持在村、社区建设“留守亲情视频热线”。大力加强文化艺术工作，完善公共文化服务设施，扩大免费开放范围；支持文化艺术精品创作，倡导全民阅读，建设书香安康；抓好“振兴汉剧”工程，办好第十六届汉江龙舟节，加强文化遗产保护与传承；推进传统媒体与新兴媒体融合发展，加大新闻原创首发力度，拓展对外文化交流，提高对外传播能力，提升安康文化软实力。加强殡葬管理，规范殡葬服务。大力发展老龄、残疾人、妇女儿童、红十字、慈善事业，让每一个困难群众都能感受到社会关爱和温暖。

  加强创新社会治理。强化政府社会治理和公共服务职能，加快由管理型向服务型转变。健全新型社区管理服务机制，发挥县区在宜居城市建设中的关键作用。支持和鼓励各类社会组织发展，充分发挥提供服务、反映诉求、规范行为等作用。创新群众工作机制和方式方法，把信访纳入法治化轨道，加强矛盾纠纷排查化解，完善多元化调解机制。加强流动人口和特殊人群服务管理。扎实推进“平安安康”建设，深入开展基层法治创建和普法宣传。加强互联网建设和管理，确保网络和信息安全。强化安全生产责任体系，实行党政同责、一岗双责、失职追责，坚决遏制重特大事故发生。依法从严打击食品药品违法犯罪，确保人民群众舌尖上的安全。强化公共安全管理，扎实做好防汛抗旱、防灾减灾工作；规范民间借贷，打击非法集资，防范金融风险；加强应急平台体系建设，提升突发事件应对能力，保障群众生命财产安全。

  办好十大民生实事。一是持续做好就业工作，推进大众创业、万众创新。新增城镇就业2万人，劳动力转移就业60万人，应届高校毕业生登记就业率90%以上。免费实施职业技能培训2万人次，发放创业小额担保贷款5亿元。与院校共同创办“创客”学院，组建创业导师团队，设立“挑战杯”创业创新奖，开展青少年科技创新大赛等活动。二是加大市政建设力度，为群众休闲健身提供更多场所。实施中心城市“三个十大项目”。围绕“突出核心”，实施环江五十里区域步行和自行车交通系统、“一江两岸”文化体育设施提升、城东大桥、汉江大剧院、东坝内环路及防洪排涝、西坝防洪保安及城堤公园、滨江大道三期、黄沟片区棚改、滨江1.2.3号地、江滩公园三期十大项目。围绕“提升江南”，实施东坝、西坝片区棚改、金州路、兴安路综合改造、瀛湖库迁路改造、西郊公园、南正街改造、移民和防洪博物馆、南环干道综合改造提升、江南“三横两纵”精细化管理示范街区创建十大项目。围绕“重心北移”，实施北环线、316国道高新段改造、长春路、创新路、红星路、长岭南路二期、张岭及中渡片区棚改、高新商业综合体、高新文化饮食、高新金融聚集区十大项目。三是继续改善办学条件，满足群众对优质教育资源的需求。完成城区教育资源整合“五校北迁”及安康中学、阳光学校、市一小、汉滨初中、兴安初中整合改建，东坝小学、江北小学、黄沟小学秋季开学招生。加快枣园九年制、西关小学、汉滨区幼儿园、市第二幼儿园等19所中小学和幼儿园改扩建。启动全市职业教育资源整合。实施260个教育项目，推进100所县城以上城区学校建设和10所普通高中改造。创造条件选派一批优秀中青年教师到全国一流高校、培训机构深造。保障进城务工子女、留守儿童、残疾儿童义务教育。整合资金实现贫困学生救助体系全覆盖，决不让一个学生因贫失学，决不让一个考上大学的学生因贫上不了大学。四是实施“健康安康”工程，改善群众就医条件。深化公立医院改革，加快组建医疗集团和医疗联合体。继续为基层培养全科医生和招录医学本科生，创造条件选派一批优秀中青年医务工作者参加国内外培训。加强医疗信息化建设，推进分级诊疗一体化服务模式，扩大居民医保直接结算覆盖范围。落实好一对夫妇可生育两个孩子政策，推进卫生、计生资源整合，配套搞好健康生育、妇幼保健、托幼等服务。建成市公共卫生服务中心、市妇幼保健院、市精神专科医院以及市人民医院门诊综合楼、市中心医院全科医生规范化培训基地、市中医院全科医生临床培养基地。全面推行新农合医疗大病保险模式，进一步减轻群众医疗负担。五是继续加大公共交通发展力度，使公共交通更加高效快捷安全。始终坚持公共交通优先发展，在科学论证基础上合理投放公交车和出租车。统筹城市公交、城际和农村客运，推进公交线路向集镇、学校、园区、景区延伸，力争年底前开通城区到县河公交。统筹规划建设公共交通场站，建成江南公交总站、高新区公交枢纽站，抓好中心城区人行天桥、公共停车位建设。加强智能交通管理，有效缓解交通拥堵。六是发展健康养老产业，建立多元化的养老服务体系。积极探索“医养结合”模式，实施好全省生态健康养老试点。大力推进社会资本举办养老机构，逐步形成以居家为基础、社区为依托、机构为支撑、具有安康特色的生态健康养老服务体系。建成市级老年公寓二期，启动吉河银龙湾、建民月河口、付家河生态养老健康城、张滩立石村和江北新义村5个生态休闲养老园区。每个县建设老年公寓，完善配套设施，新建15个社区老年人日间照料中心和100个农村幸福院。七是实施安居工程，进一步改善城镇中低收入家庭和农村群众居住环境。加大城中村、棚户区改造力度，解决好这些群众的住房困难问题。提高保障性住房竣工率、入住率，保证分配公开、公平、公正，使真正需要帮助的低收入住房困难群众受益。加大投入力度，加快公租房建设步伐。围绕房地产“去库存”，打通商品房与保障性住房转换通道。继续推进避灾扶贫搬迁，完成2.7万户10.2万人搬迁安置。八是实施文体惠民工程，满足群众日益增长的精神文化需求。深入推进中心城市“文化大本营”工程和基层综合文化服务中心建设。加快汉江大剧院、香溪书院、市体育馆建设，安康学院院地共建图书馆、“藏一角”、移民和防洪博物馆建成。实施移民搬迁有线电视户户通工程。加快建设县区标准足球场。实施优秀中青年作家“十强计划”、优秀文化人才“百优计划”。创造条件，推动各类院校体育设施一直向社会公众开放。九是构建覆盖城乡的救助体系，持续关注生活困难家庭。进一步做好对残疾人、失独家庭、空巢老人以及农村留守儿童、留守老人、留守妇女的关爱工作。加强残疾人职业和实用技术培训，建设市级残疾人康复中心。将孤寡老人和丧失劳动力残疾人全部纳入低保，将贫困人口全部纳入重特大疾病救助范围，加强对计划生育特殊困难家庭的扶助关怀和精准帮扶，编织一张兜住困难群众基本生活的安全网。十是强化社会治安管理，增强群众安全感和满意度。推进社会治安视频监控系统建设，新建1300个视频监控，努力实现县城以上视频监控无盲区、无死角；创新完善街面巡防机制，织密环安防控圈，夯实基层基础；强化出租房和流动人口管理，推进社区联防和群防群治，构建“空中有监控、地面有巡逻、出入有卡点、社区有联防”的立体化治安防控体系。始终保持对违法犯罪的高压态势，严厉打击“盗抢骗”、“黄赌毒”、“食药环”等违法犯罪，继续开展“打五霸”和“破小案、暖民心”活动，不断提高人民群众对社会治安的满意度。

  同时，认真做好国防动员、双拥共建、国家安全、民族宗教、优抚安置、人民防空以及统计、移民、对台、史志、档案等工作，支持工会、共青团、妇联、文联、残联、侨联、工商联、红十字会等群团组织发挥更大作用。

  （七）坚持从严治政，建设人民满意政府

  深入推进依法行政。坚持把宪法和法律作为政府工作的根本准则，推动尊法学法用法守法成为常态、依法全面履行职能职责成为常态、用法治方式推进改革发展成为常态。完善依法决策机制，严格执行法定决策程序，重大决策深入论证、公开征求意见，推进全市行政机关法律顾问全覆盖。完善政府立法机制，提高政府立法质量。深化行政执法体制改革，推进执法力量下沉和综合执法，坚决纠正执法不严、不公、不规范、不文明现象。加大政务公开力度，拓展政务中心服务领域，推行电子政务和网上办事。自觉接受人大、政协及社会各界监督，注重发挥新闻媒体和网络作用，让行政行为在阳光下运行，充分体现人民意愿。

  力倡厉行“三严三实”。把“三严三实”贯穿政府工作全过程，坚持目标导向和问题导向相统一，进一步强化责任、担当、落实，说了算、定了干，敢于担当、积极作为，以开局就是决战、起步就是冲刺的劲头，以逢山开路、遇河架桥的勇气，以偏向虎山行、敢啃硬骨头的魄力，立下愚公志，实现新跨越。深化政府管理改革创新，持续推进简政放权，强化行政效能监察，完善绩效管理和考核评价体系，跟踪督办重大决策，持之以恒改进作风，敬终如始查纠“四风”，坚决惩治乱作为、庸懒散、办事难等作风问题。始终把增进群众福祉作为最大责任，用心用情做好惠民生、聚民心的工作，继续实行重点工作和民生实事公开承诺制，进一步提升政府公信力。畅通联系和服务群众渠道，把全市人民的关注点引导到发展上，唱响主旋律、发出好声音、凝聚正能量，创造心齐、气顺、劲足的发展环境，依靠全市人民的热情参与加快改革发展。

  持续推进廉政建设。坚决贯彻中、省和市委从严治党、从严治政的部署要求，夯实“一岗双责”主体责任，做到抓发展必抓廉政、管行业必管行风，对各类违法违纪行为零容忍、严查处，更加重视解决基层特别是发生在群众身边的不正之风和腐败问题，不断增强群众对正风肃纪的获得感。加强行政权力运行监督，完善重点领域、关键岗位廉政风险防控机制，对公共资金、国有资产、国有资源和领导干部经济责任情况实行审计全覆盖。加快建设公共资源交易平台，促进工程招投标、土地招拍挂、政府采购等更加公开透明、规范高效。继续开展土地管理、工程建设、资源开发、产权转让、政府采购等领域的专项治理，严肃查处违纪违法行为。加强政府系统理论学习和党规党纪教育，补足精神之钙、强化自律之基，始终做到心中有党、心中有民、心中有责、心中有戒。正确处理严管干部与爱护干部的关系，理直气壮地为改革者“鼓劲”、为担当者“撑腰”，既旗帜鲜明地支持担当者、保护担当者、褒奖担当者，又旗帜鲜明地与不作为、乱作为“较劲”，让那些庸者、自毁者受到惩处，让奋发有为的干部得到激励，努力营造干部清正、政府清廉的良好政治生态。

  各位代表，时代赋予我们追赶超越的历史机遇，人民满怀对更加美好生活的期待。让我们在中共安康市委的坚强领导下，进一步解放思想、振奋精神、激发干劲、砥砺前行，不辜负大有作为的时代，不辜负全市人民的重托，为实现“十三五”规划目标、建设美丽富裕新安康而不懈奋斗！
